# A polymerase engineered for bisulfite sequencing

**DOI:** 10.1093/nar/gkv798

**Published:** 2015-08-13

**Authors:** Doug Millar, Yonka Christova, Philipp Holliger

**Affiliations:** 1 Genetic Signatures, Level 9, Lowy Packer Building 405, Liverpool Street, Darlinghurst 2010, Sydney, Australia; 2 MRC Laboratory of Molecular Biology, Francis Crick Avenue, Cambridge Biomedical Campus, Cambridge CB2 0QH, UK

## Abstract

Bisulfite sequencing is a key methodology in epigenetics. However, the standard workflow of bisulfite sequencing involves heat and strongly basic conditions to convert the intermediary product 5,6-dihydrouridine-6-sulfonate (dhU6S) (generated by reaction of bisulfite with deoxycytidine (dC)) to uracil (dU). These harsh conditions generally lead to sample loss and DNA damage while milder conditions may result in incomplete conversion of intermediates to uracil. Both can lead to poor recovery of bisulfite-treated DNA by the polymerase chain reaction (PCR) as either damaged DNA and/or intermediates of bisulfite treatment are poor substrate for standard DNA polymerases. Here we describe an engineered DNA polymerase (5D4) with an enhanced ability to replicate and PCR amplify bisulfite-treated DNA due to an ability to bypass both DNA lesions and bisulfite intermediates, allowing significantly milder conversion conditions and increased sensitivity in the PCR amplification of bisulfite-treated DNA. Incorporation of the 5D4 DNA polymerase into the bisulfite sequencing workflow thus promises significant sensitivity and efficiency gains.

## INTRODUCTION

**Figure 1.**
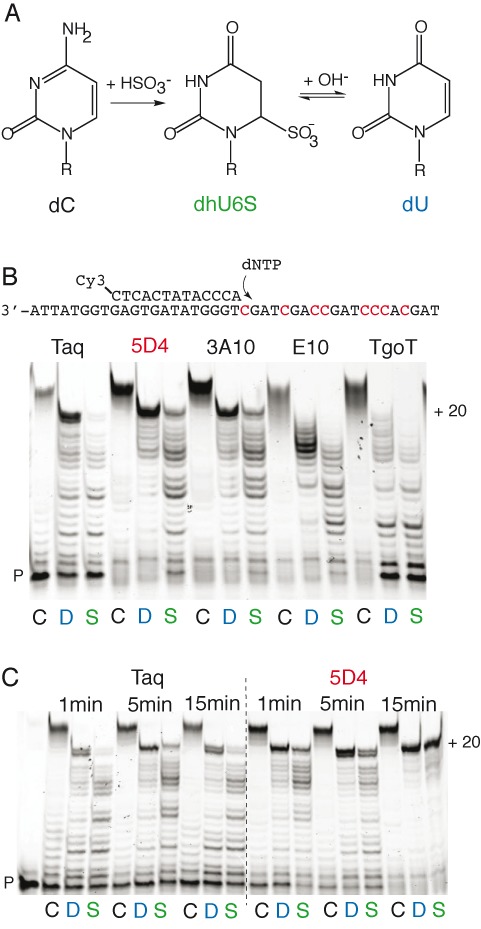
DNA modification and bypass during bisulfite treatment. (**A**) Scheme of the chemical reaction of cytidine: Treatment with bisulfite generates the non-aromatic, non-planar 5,6-dihydrouridine-6-sulfonate (dhU6S), which decomposes to uracil upon treatment with base (and heat). (**B**) Primer extension activity of different polymerases (Taq, 5D4, 3A10, E10, TgoT) on template T1 either unmodified (C), bisulfite-treated and desulfonated (Reagent 1, 80°C, 20 min) (converting dC to dU) (D) or bisulfite-treated (Reagent 1) but not desulphonated (converting dC to dhU6S) (S). Polymerases 5D4 and 3A10 are able to generate full-length (+20) products even from the non-desulphonated template (S). (**C**) Time-course comparison of primer extension activity of Taq and 5D4 on T1 either unmodified (C) or bisulfite-treated with (D) or without desulphonation (S). (P: primer).

The bisulfite treatment of nucleic acid was first described 45 years ago (1) and has become a key method in epigenomics. Treatment of DNA with sodium bisulfite results in the deamination of cytosine to a 5,6-dihydrouracil 6-sulphonate (dhU6S) intermediate (Figure 1). Further treatment of the uracil sulphonate with strong alkali is necessary to complete the reaction resulting in the formation of a uracil residue (dU) at all sites in the DNA that were originally occupied by cytosine. 5-Methylcytosine (5mC) on the other hand is resistant to the treatment and remains intact after completion of the reaction. Thus, bisulfite chemistry allows the determination of the positions of the epigenetic marker 5mC in DNA by mapping the conversion of bases read as dC before and after bisulfite treatment. In 1992 a paper was published describing the use of the bisulfite reaction for sequencing methylated cytosines in mammalian DNA (2) for the first time. Since then the bisulfite reaction has become the method of choice in epigenetics and epigenomics for determining the methylation profiles of genes of interest.

Numerous variations of the bisulfite method such as Methylation-Specific PCR (MSP) (3), Combined Bisulfite and Restriction Analysis (COBRA) (4), Single Nucleotide Primer Extension (SNuPE) (5) and micro-array-based approaches have been developed, demonstrating the universal utility of the bisulfite reaction for the detection of 5mC in DNA. The bisulfite method has also been used in combination with next-generation sequencing to produce the first complete human epigenome at single base resolution (6) and has yielded valuable information regarding the role of both CpG and CNG methylation in gene regulation. More recently, combination of the bisulfite chemistry with oxidation and reduction steps have been used to map other epigenetic marks including 5hmC (5-hydroxy-methyl cytosine) and 5fC (5-formyl-cytosine) (7,8). Real-Time sequencing (SMRT) (9), as well as Nanopore sequencing (10), hold the promise of a direct readout of epigenetic marks like 5mC, 5hmC and 5fC, but current levels of accuracy suggest that for many applications bisulfite sequencing will remain the standard method for some time.

Early on in the development of bisulfite method, it was discovered that sulphonated DNA was an exceedingly poor template base for DNA polymerase I (11), which was unable to extend past the sulphonate adduct. Termination may be caused by both the presence of the bulky and charged sulphonate group in the C6 position as well as the deviation from planarity in the non-aromatic ring system of the 5,6-dihydrouracil-6-sulphonate (dhU6S) intermediate, which results in distortions of DNA geometry and reduced stacking interactions. DNA binding proteins such as RecA and SSB can result in partial read-through indicating that the binding of these proteins to upstream template regions can relax some of the distortions and allow the polymerase to read through the lesion site (12). Removal of the sulphonate group by treatment with base allows polymerases to copy the modified DNA strands, due to the conversion of the distorted dhU6S structure to the planar cognate uracil but at the cost of DNA damage and sample loss through the harsh treatment. While the bisulfite reaction itself has become the gold standard in epigenetic research, it has been shown that bisulfite treatment can result in up to 96% degradation of the input DNA (13). Such DNA loss is severe, hampering the application of the bisulfite method when the starting DNA is limiting such as in stem cell research, microdissected DNA and archival tissue samples. It has been found that the desulphonation step is possibly the most damaging step in the bisulfite treatment of DNA. The desulphonation step generally requires not just strongly basic conditions but also elevated temperatures for quantitative adduct removal, accelerating DNA damage through depurination and base oxidation. Consequently, amplification of bisulfite-treated DNA has been found to be considerably more challenging than non-bisulfite-treated DNA and can result in significant polymerase chain reaction (PCR) bias, which can severely affect methylation estimates (14). This problem arises from a number of contributing factors, which may include mispriming (due to the simplification of the genetic alphabet from four to three bases), a difficulty in copying both dU-rich DNA sequences and heavily modified DNA and/or irreversible damage or fragmentation of some DNA templates rendering them unamplifiable.

The universal enzyme of choice for the amplification of bisulfite-treated DNA since the first published studies has been *Thermus aquaticus* DNA polymerase (Taq). Other PCR enzymes, such as archaeal polymerases are unable to efficiently copy bisulfite-treated DNA due to the stalling triggered by template uracil (15). Mutants such as PfuV93Q have been generated that reduce the stalling of the polymerase at uracil-containing DNA but these enzymes still appear to be suboptimal for the amplification of bisulfite-treated templates. We therefore reasoned that, if a polymerase could be engineered specifically for the efficient amplification of bisulfite-treated DNA, significant gains in efficiency and sensitivity in the genome-wide determination of CpG methylation status could be obtained. Such a polymerase ideally could bypass template damage as well as bisulfite intermediates and template deoxyuracil and thus efficiently copy even a partially desulphonated template.

Here we describe and characterize the chimeric polymerase (5D4), previously isolated for its ability to utilize unnatural hydrophobic base analogues (16), as a first example of such an engineered polymerase. 5D4 demonstrated not only significantly enhanced DNA synthesis efficiency on bisulfite-treated DNA and enhanced bypass of dhU6S adducts in bisulfite-treated non-desulphonated DNA but also improved sensitivity in PCR amplification of bisulfite-treated DNA or uracil-containing DNA templates in general.

## MATERIALS AND METHODS

### Polymerases

Polymerases 3A10 (17) and 5D4 (16) were obtained by compartmentalized self-replication (CSR) selection (18) and polymerase (19) E10 by short-patch CSR selection (20) as described. TgoT comprises a A485L mutation of the Tgo DNA polymerase. Taq DNA polymerase (SuperTaq) was obtained from HT Biotechnology (Cambridge) and Q5 polymerase from New England Biolabs. Expression of polymerases for characterization was as described (17,19) using a 16/10 Hi-Prep Heparin FF Column (Amersham Pharmacia Biotech). Polymerase fractions eluted around 0.3M NaCl and were concentrated and dia-filtered into 50 mM Tris pH 7.4, 1 mM Dithiothreitol (DTT), 50% glycerol and stored at −20°C. 5D4 polymerase is available from the authors upon request.

### Fluorescent primer extension analysis

A total of 100 pmol of template primer, 5 pmol of target oligonucleotides, 50 μM dNTP's, 1x Taq polymerase buffer in a final volume of 48 μl were heated at 95°C for 1.5 min then cooled to 40°C and pre-incubated at 55°C. One unit of Taq polymerase or 1 μl of purified 5D4 polymerase was then added to each reaction and the components incubated at 55°C. Reactions were stopped by the addition of an equal volume of stop solution (8M Urea, 50 mM ethylenediaminetetraacetic acid (EDTA)). Before being loaded onto polyacrylamide gels, the reactions were denatured at 95°C for 2 min then placed on ice. Eight microlitres was loaded per well and products resolved using denaturing polyacrylamide gel electrophoresis (8M Urea, 20% acrylamide, 25 W for 4.5 h) and visualized using a Typhoon imager.

### Bisulfite treatment

Two micrograms of genomic DNA (Promega) were dissolved in water (Sigma) to a final volume of 18 μl and 2 μl of 2M NaOH added and the sample mixed well by pipetting. The sample was incubated at 37°C for 15 min to denature the DNA strands. A total of 220 μl of 3M sodium bisulfite was added and the sample mixed briefly then heated at 80°C for 45 min. The samples were desalted using the MethylEasy™ Xceed kit (Human Genetic Signatures, Sydney, Australia) according to the manufacturers instruction. The DNA was eluted in a final volume of 20–100 μl.

### PCR amplification

Amplification mixes consisted of 1x SuperTaq buffer (HT Biotech, Cambridge, UK), 0.5 μl 5D4 Polymerase, 100 ng primers, 50 μM dNTPs and 2 μM Syto 9 (Life Technologies). For comparison with regular Taq polymerase PCR reactions were also set up using 1x Promega master-mix again using 100 ng of both forward and reverse primer and Syto 9 (according to the manufacturers instructions). Thermal cycling consisted of 1 cycle 95°C for 2 min and 45 cycles of 95°C for 5 s, 55°C for 30 s, 68°C for 30 s unless otherwise stated. Products were amplified using a BioRad CFX real-time PCR instrument (BioRad) collecting the data after the final extension step using the 6-Carboxyfluorescein (FAM) channel. After completion of the cycling reaction PCR products were confirmed on a 2% E-gel (Invitrogen) according to the manufacturers instructions and photographed using a Kodak gel Doc instrument under UV transillumination.

### The effect of uracil on amplification of bisulfite treated templates

Two genomic loci were selected to determine the effect of uracil incorporation by Taq polymerase in a PCR amplification reaction using bisulfite treated genomic DNA. The two regions selected, *cxcl2* (320 bp amplicon) and *dbccr1* (230 bp amplicon), have a similar A content of 15.3 versus 16.9% respectively but a C content of 26.6 versus 18.7%. Genomic DNA was bisulfite converted and amplified using a series of PCR reactions containing 10 µM dATP, dCTP, dGTP each in which dTTP was substituted with dUTP at the following concentrations dTTP 10 µM, 8 mM dTTP/2 µM dUTP, 6 µM dTTP/4 µM dUTP, 4 µM dTTP/6 µM dUTP, 2 µM dTTP/8 µM dUTP, 1 µM dTTP/9 µM dUTP and finally 0.5 µM dTTP/9.5 µM dUTP. Amplification was carried out using a BioRad CFX real-time PCR instrument as previously described.

### The effect of cytosine content on amplification efficiency

An in house plasmid (pMUP) was used to determine the effect of C content on the amplification efficiency of 5D4 and Taq polymerase. The plasmid contained two distinct regions one of around 600 bp that had a C content of 11.9% (72 cytosine residues) and a higher C content region of 700 bp that had a C content of 23% (161 cytosine residues) (Figure 3). Three primer sets were prepared for each region (Table 1).

**Table 1.** Primer combinations, amplicons and C content (%)

Low C content region				High C content region			
Primers	Amplicon	C residues	%C	Primers	Amplicon	C residues	%C
F1/R1	160 bp	20	12.5	F1/R2	348 bp	101	29
F1/R3	244 bp	23	11.1	F1/R4	454 bp	120	26.4
F1/R6	497 bp	53	10.7	F1/R6	624 bp	152	24.4

About 10^6^ copies of the pMUP plasmid were bisulfite treated in a final volume of 220 μl using the MethylEasy™ Xceed kit according to the manufacturers instructions. A total of 2 μl of the purified plasmid was amplified in a PCR reaction mix consisting of 1x GoTaq flexi buffer, 10 µM dNTP's, 2 mM MgCl_2_ and either 2.5U of GoTaq Flexi (Promega) or a GoTaq/5D4 polymerase blend of 10:1, 5:1 and 1:1. PCR was carried using 35 cycles of 95°C for 5 s, 55°C for 10 s and 68°C for 10 s. Amplicons were resolved using 2% precast E-gels (Life Technologies) according to the manufacturers instructions.

### The effect of increasing PCR extension time on desulphonated/sulphonated templates

To further determine the effect of cycling times on the ability of 5D4 to amplify both desulphonated and fully sulphonated templates the primer set F1/R2 that amplifies the high C content pMUP region was used. About 10^6^ copies of the plasmid were bisulfite-treated in duplicate using the MethylEasy™ Xceed kit according to the manufacturers instructions (Human Genetic Signatures, Sydney, Australia). One plasmid was desulphonated as per manufacturers instructions while the other plasmid was resuspended in the same volume of distilled water to generate a fully sulphonated plasmid template. PCR premixes were prepared as previously described containing either Taq polymerase alone or a 10:1, 5:1 and 1:1 Taq polymerase/5D4 blend. PCR was carried out as above with the addition of an extended first cycle of PCR where the extension time was increased to either 5 or 10 min for that cycle only then cycled for the remaining 34 cycles as previously described. The efficiency of the PCR amplification was determined by the addition of Syto 9 (Life Technologies) according to the manufacturers instructions using a BioRad CFX realtime thermal cycler.

### Amplification of human genomic loci using 5D4

Twenty-four individual human genomic target regions were chosen at random to determine the efficiency of 5D4 blends compared to reaction performed using Taq polymerase alone. The target regions were; *abcb5, brca1, casp8, cftr, cxcl2, bcl2, dapk1, dbc, dlk1, erbb4, hoxa5, hoxa11, hic1, igfbp3, igf2, magea3, mgmt, muc1, psen1, rassf1, rfc1, rarb and serpina5*. Fully nested primers that amplify a portion of the promoter region of each target were used in the amplification reactions (see Supplementary Information and Table S2 for full details of the primer sequences used). A two round PCR reaction was performed using a 5:1 Taq polymerase: 5D4 and a 10:1 Taq polymerase: 5D4 blends and comparing these to Taq polymerase. The second round PCR was performed using standard Taq polymerase.

First round PCR amplification mixes consisted of 1x SuperTaq buffer (HT Biotech, Cambridge, UK), 2.5 μl Taq polymerase : 5D4 Polymerase blend, 100 ng primers, 50 μM dNTPs and 2 μM Syto 9. For comparison with regular Taq polymerase, PCR reactions were also set up using 1x Promega master-mix again using 100 ng of both forward and reverse primer and 2.5 μM Syto 9. Two microlitres of first round material was transferred to a second round amplification mixes consisted of 1x Promega master-mix again using 100 ng of both forward and reverse primer and Syto 9 as previously described. Thermal cycling consisted of 1 cycle 95°C for 2 min the 30 cycles of 95°C for 5 s, 55°C for 10 s, 68°C for 10 s. Products were amplified using a BioRad CFX real-time PCR instrument, collecting the data after the final extension step using the FAM channel and collection step of 5 s at 75°C. After completion of the cycling reaction PCR products were confirmed on a 2% E-gel (Invitrogen) according to the manufacturers instructions and photographed using a Kodak Gel Doc instrument under UV transillumination.

### Illumina sequencing

Human genomic DNA (Promega) or genomic DNA isolated from LNCaP prostate cancer cell line using DNeasy Blood and Tissue kit (Qiagen) were subjected to bisulfite conversion by MethylEasy™ Xceed kit (Human Genetic Signatures, Sydney, Australia) following the manufacturer instructions. Primers were designed to amplify the BS converted promoter regions of four genes - *prkcdbp, ptgs2, ezh2* and *dab2ip* (Supplementary Table S5).

Four amplicons were generated for each gene: two with each Taq or 5D4/Taq blend, using as templates either human genomic DNA (Promega) or LNCaP prostate cancer cell line genomic DNA. Fully nested PCR was used to obtain each amplicon. First PCR was performed either with Taq or Taq/5D4 blend using 2 μl of primers, 1 μl of template in 25 μl reaction under following conditions: 3 min at 95°C; 1 min at 95°C, 2 min at 50°C, 2 min at 72°C for 20 cycles; 10 min at 72°C. The second PCR used 2 μl from the first PCR as a template and was performed with GoTaq (Promega) under the following conditions: 2 min at 95°C; 30 s at 95°C, 30 s at 52°C, 30 s at 72°C for 30 cycles; 10 min at 72°C. To prepare the amplicons for Illumina sequencing we have used sequencing adaptors and index sequences based on Illumina P3 and P5 primers. Three random nucleotides were inserted between the adaptor sequence and the amplicon specific sequence to facilitate the cluster identification during Illumina sequencing. The full list of adaptors is presented in Supplementary Table S6. The PCR reactions consisted of 1 μl template, 0.5 μM primers and 12.5 NEBNext High-Fidelity 2X PCR Master Mix (New England Biolabs). Cycling conditions were 2 min at 98°C; 10 s at 98°C, 30 s at 55°C, 30 s at 72°C for 20 cycles; 5 min at 72°C. After gel purification, the PCR products were quantified by qPCR using KAPA Library quantification kit. All amplicons were pooled in equimolar ratios and sequenced using an Illumina v-3 reagent kit (150 cycles) at 12 pM concentration, supplemented with 15% PhiX.

The sequencing reads in fastq format were barcode split and processed using www.galaxyproject.org. Burrows-Wheeler aligner (BWA; version 0.7.7) was used to align the sequencing reads to the genomic sequence of each amplicon, BS treated *in silico*. Alignments were converted from sequence alignment map (SAM) format to sorted, indexed binary alignment map files using SAMtools version 0.1.19; http://sourceforge.net) and the base calls were made by mpileup command. Post-variant calling analyses was performed using a custom MATLAB script (kindly provided by A. Morgunov, available upon request). In calculating the bisulfite conversion, only Cs outside CpG were taken into account. When determining the error rate, C to T errors were excluded from the calculation, to avoid a potential bias of BS conversion or methylation heterogeneity. When the breakdown of error rates by individual nucleotides was determined, only the error rates for G, A and T in the original (untreated) genomic sequence were calculated, as the uncertainty whether C has been converted to T would otherwise introduce unpredictable error in the calculations.

## RESULTS

We sought to discover a polymerase that could amplify bisulfite-treated DNA. We initially screened polymerases generated in our laboratory by directed evolution using CSR (18) from both the polA- and polB-family, using a primer extension assay on bisulfite-treated DNA templates. We designed a 20 nt DNA template comprising eight deoxycytidine dC groups (40% C content over 20 bases) containing three single dCs as well as one dC_2_ duplet and one dC_3_ triplet each, but lacking dCs in the primer binding site (T1, Supplementary Table S1). Thus bisulfite treatment of this template DNA would convert those dCs to deoxyuridine (dUs) via a 5,6-dihydrouridine-6-sulfonate (dhU6S) intermediate (Figure 1A), without abrogating primer binding, enabling an accurate comparison of the ability of polymerases to read through the dC-rich template sequence after bisulfite treatment.

We tested polymerase mutants from the polB family including i.a. E10, a mutant of Pfu DNA polymerase capable of efficient PCR amplification of full Cy3,5-dC substituted DNA (19) and TgoT, a variant of the DNA polymerase from *Thermococcus gorgonarius* with a Therminator mutation (21). We also tested polymerase mutants from the polA family, including - apart from Taq DNA polymerase - 3A10 (17), selected for an ability to bypass template lesions and 5D4 (16), selected for an ability to utilize hydrophobic base analogues. All the polB family polymerases tested were poor at copying the bisulfite-treated template even though they contained the V93Q mutation. However, among polA-family polymerases, two evolved polymerases 5D4 and 3A10, showed significant promise as a candidate for enhanced activity on bisulfite-treated templates (Figure 1B) yielding full-length (+20) extension products on both desulfonated template (D), in which dC is mostly converted to dU as well as, remarkably on non-desulphonated template (S), in which the majority of dC remained as the dhU6S intermediate. Indeed, 5D4 could yield full-length extension products on both templates in <1 min (Figure 1C).

These results suggest that 5D4 as well as having an enhanced ability to utilize deoxyuracil (dU) as a template base can also utilise the sulphonated uracil adduct dhU6S as a template base albeit at a reduced efficiency compared to dU. In addition the results suggest that bisulfite-treated DNA is not copied as efficiently as non-treated DNA and that bisulfite templates take longer for the polymerase enzymes to copy. This may be due to stalling at dU residues, as well as due to (some degree at least) the potentially incomplete removal of sulphonate groups from all dhU6S adducts or the potential presence of other forms of DNA lesions such as abasic sites and oxidative lesions.

**Figure 2.**
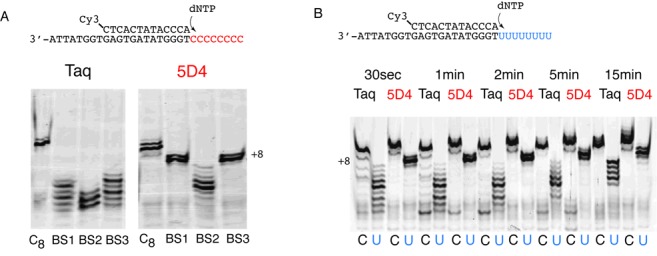
Sulphonate conversion and template base effects on polymerase activity. (**A**) Activity of Taq polymerase and 5D4 polymerase in copying a polyC (dC_8_) (Template T2, 5 min extension) either unmodified and bisulfite-treated and desulphonated using two different approaches (BS1, 3) or not desulphonated (BS2). Only 5D4 can successfully bypass the bisulfite-treated polyC stretch and generate full length (+8) product but only after (at least partial) desulphonation. (**B**) Comparison of polymerase activity of Taq and 5D4 on a dC_8_ (T2) compared to a dU_8_ (T3) template for different extension times (0.5–15 min). Clearly, dU is a poor template base for Taq polymerase. Please note that dU_8_ and dC_8_ template extension products (dA_8_ and dG_8_) display slightly different mobility in a 20% urea-polyacrylamide gel due to the different base compositions as can also be seen in Figure 1B and C.

For further characterization we concentrated on the superior 5D4 polymerase. 5D4 is a chimeric polymerase, comprising segments from the *T. aquaticus* (Taq) and *T. thermophilus* (Tth) DNA polymerases as well as 14 additional mutations (16). It was originally isolated for its generic ability to utilise nucleic acids comprising hydrophobic non-hydrogen bonding base analogues such as 5-nitroindole as a substrate (18). To characterize 5D4 in more detail and compare its activity on bisulfite-treated DNA to that of Taq DNA polymerase, we first examined the effect of bisulfite treatment of template DNA on the efficiency of primer extension using a template comprising a 5′ dC_8_ stretch (T2, Supplementary Table S1), which after bisulfite treatment and complete desulphonation would convert to a dU_8_ sequence. To investigate the effect of potentially incomplete desulphonation on polymerase extension, we investigated polymerase read-through of the bisulfite-treated T2 template subjected to three different desulphonation conditions of varying efficiency (and harshness). The first was the standard method of buffer 1 (R1: 10 mM CAPS/0.1 mM EDTA pH 10.5) coupled with heat treatment at 80°C for 10 min (BS1), the second (BS2) used R1 without any heat treatment and the third (BS3) was resuspended in more basic buffer 2 (R2: 10 mM CAPS/0.1 mM EDTA pH 11.5) again without heat treatment (with the higher pH of the buffer 2 promoting desulphonation). The commonly used method (BS1) uses harsh conditions (both high pH and heat) to achieve (next to) complete desulphonation, at the cost of increased DNA damage. The other two variations used here either use the same reagent as in method 1 but without any heat treatment (achieving only incomplete desulphonation, BS2) or an alternative low temperature regime (BS3) with an even higher pH (11.5) (yielding more complete desulphonation than BS2). If 5D4 was superior to Taq then improved extension should be observed in all lanes especially BS2, where DNA would only be very partially (if at all) desulphonated. We thus processed bisulfite-treated dC_8_ template using methods BS1–3 and compared the ability of Taq and 5D4 DNA polymerases to copy each of the prepared templates. As can be seen the standard bisulfite-treated template (BS1) and presumed dU_8_ sequence serves as a poor template for Taq polymerase even when the uracil has been fully desulphonated (Figure 2A, left panel) and no dhU6S groups should remain. 5D4 on the other hand produces full-length extension products without any significant pausing when assayed on the same templates under the same conditions (Figure 2A, right panel). Both polymerases struggle to copy the non-desulphonated, presumed dhU6S_6_ sequence (BS2), with 5D4 superior to Taq. Desulphonation at higher pH (BS3) yields similar extension results to standard bisulfite treatment (BS1) for both polymerases (Figure 2A, right panel). Furthermore, 5D4 correctly inserts dA both opposite template dU as well as opposite template dhU6S (Supplementary Figure S1).

We wondered if the poor efficiency of Taq extension on the presumed dU_8_ template was due to incomplete removal of the sulphonate groups from dhU6S or potentially due to dU itself being a poor template base. We therefore prepared a synthetic template in which the dC_8_ stretch was replaced by dU_8_ (T3, Supplementary Table S1). Indeed, when we compared 5D4 and Taq using both the dU_8_ and the dC_8_ template without bisulfite treatment, we found that while 5D4 completely copied past all dU residues in the template in 30 s, Taq exhibited significant pausing. Even after 15 min of incubation Taq polymerase had still not yielded a quantitative full-length extension product from the polydU-template and still showed multiple termination bands (Figure 2B).

In summary these results suggest that 5D4 displays a significantly superior capability compared to Taq polymerase to copy bisulfite-treated DNA. Part of this effect is due to the fact that dU itself appears to be a rather poor template base for Taq polymerase in particular in homopolymeric runs (Figure 2B). Together with difficulties in bypassing template lesions and residual dhU6S adducts this is likely to contribute to a reduced efficiency in PCR for amplifying bisulfite-treated DNA for Taq DNA polymerase.

To determine the potential inhibitory effect of dU on PCR of bisulfite-treated DNA with Taq DNA polymerase, we examined PCR efficiency by qPCR using dNTP mixes with varying ratios of dTTP and dUTP (from 0% dUTP to 100% dUTP (resulting in varying amounts of template dU in later rounds of PCR)) and amplifying a bisulfite-treated (BS1) 0.32 kb fragment from the chemokine (C-X-C motif) ligand 2 (*cxcl2*) and a bisulfite-treated (BS1) 0.23 kb fragment from the ‘deleted in bladder cancer 1′ (*dbccr1*) promoter region (Supplementary Figure S2). The two genomic regions varying in total C content (26.6 versus 18.7% respectively thus have a differing dU content prior to amplification). Incorporation of dUTP in the early cycles generates mixed dU/dT templates. Taking advantage of the iterative nature of PCR, which can amplify even subtle differences, we would expect a significant drop in PCR efficiency using dUTP, if dU is a poor template base. Indeed, we find that dUTP cannot efficiently substitute for dTTP in PCR of both target regions using Taq polymerase. Even at a dTTP/dUTP ratio 2:8 the amplification efficiency was reduced by nearly 1000-fold, although the effect was highly sequence dependent. While complicated by potential effects of dUTP as a substrate, these data point to dU being a poor template base for Taq. The sequence dependence of the observed effect also suggests that there may be a certain uracil content threshold or length of homopolymeric dU runs above which this inhibitory effect begins to degrade PCR efficiency of Taq polymerase potentially leading to biases in the amplification of bisulfite-treated DNA, as had been previously observed (14).

**Figure 3.**
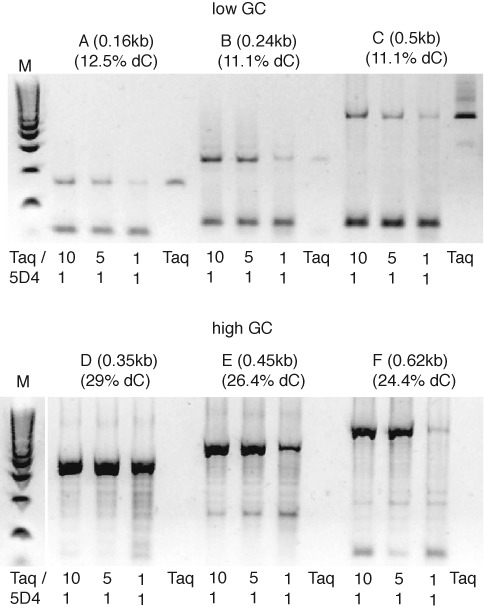
PCR amplification of bisulfite-treated plasmid templates. PCR amplification of bisulfite-treated high GC and low GC content templates ranging from 200–600 bp in size using fully desulphonated templates and three different 5D4/Taq blends (1/10, 1/5, 1/1) with progressively lower Taq content and Taq alone on low dC content plasmid regions (top panel) and high dC content plasmid regions (bottom panel). On templates with low dC content (and hence lower levels of dU and residual dhU6S adducts post bisulfite treatment and desulphonation) either Taq or Taq/5D4 polymerase blends with a high amount of Taq perform best. In contrast on the higher dC content templates only blends containing 5D4 yield amplicons with Taq/5D4 blends (10/1; 5/1) superior to 5D4/Taq 1/1 blend, while Taq alone does not yield any amplification products. Thus only 5D4/Taq blends are able to copy the high GC content templates indicating that the blended enzymes are more efficient at copying templates containing sporadic dUs (and dhU6S adducts) and dU homopolymer stretches. Low molecular weight bands result from primer-dimer formation. (M: E-Gel^®^ Low Range Quantitative DNA Ladder).

Having established the striking ability of 5D4 to efficiently bypass and copy synthetic templates containing sporadic or homopolymeric stretches of dU (desulphonated dC) or non-desulphonated dhU6S (Figures 1 and 2), we sought to compare the ability of 5D4 and Taq to PCR amplify bisulfite-treated (BS1) target regions starting with plasmid sequence segments containing both low (A–C) and high (D–F) dC content target inserts (Figure 3). While Taq polymerase alone was capable of good amplification of two out three (A and C) of the low GC content regions (and weak amplification from low GC region B) (Figure 3, top panel), on its own it yielded no amplicons at all of the high GC content regions (D–F). In contrast, different blends of 5D4 and Taq yielded robust amplification of all three high GC content regions (D–F) (Figure 3 bottom panel) and equal or stronger amplicons of the low GC regions (A–C). These results demonstrate clearly that 5D4 has a greatly enhanced ability to PCR amplify bisulfite-treated templates with high dC content compared to Taq polymerase using the standard bisulfite treatment method (BS1). Regions containing low dC content are not as strongly affected, probably due to a decrease in enzyme stalling at dU resulting from a reduced likelihood of dU homopolymer runs. Most beneficial is a blend of both Taq and 5D4 DNA polymerase at a ratio of 10/1 or 5/1, where the overall superior PCR performance of Taq is combined with the superior ability of 5D4 to traverse template adducts and lesions as well as dU homopolymer runs.

To further characterize the ability of 5D4 to amplify regions of high dC content, a 348 bp fragment from the high dC content region D (Figure 3) containing 101 dC (29% dC) was targeted under four independent desulphonation conditions (Supplementary Figure S3) including the standard BS1 method combined with a higher than normal heat treatment at 95°C for 10 min to expedite as complete as possible desulphonation (BS1T), BS2 (no heat treatment), BS3 (high pH) and water (no desulphonation control). As can be seen 5D4 yields strong amplification band under all conditions except the water control, while Taq produces only weak amplification signals even when harsh conditions are used to effect near complete desulphonation (BS1 and 3). 5D4, in contrast, yields strong amplification bands even in sample 2, where due to mild BS2 treatment, only incomplete desulphonation occurs. Indeed, 5D4 even yields an amplification signal (although weak) for the fully sulphonated DNA (lane 4) (Supplementary Figure S3), indicating that 5D4 has some ability to amplify non-desulphonated templates (containing up to 29% dhU6S adducts).

**Figure 4.**
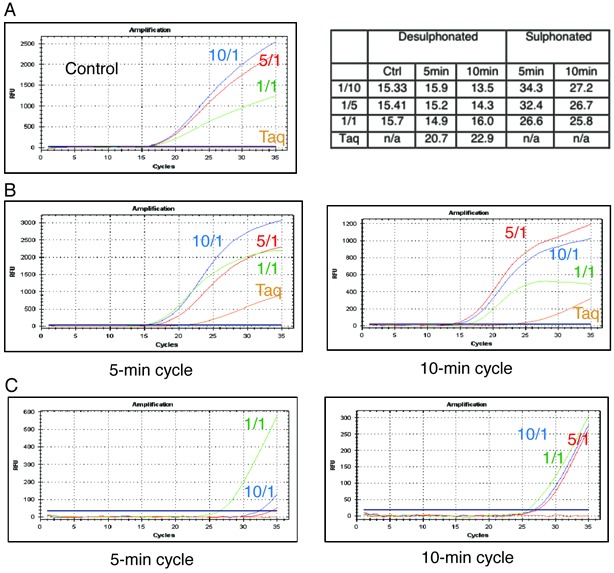
qPCR of bisulfite-treated plasmid templates. qPCR of high CG content template (0.62 kb, Figure 3 region F) using both fully desulphonated (**A**, **B**) and sulphonated templates (**C**) with Taq alone (orange) as well as three different 5D4/Taq blends (1/10 (blue), 1/5 (red), 1/1 (green)). qPCR Ct values for these experiment are tabulated (top right panel). (A) qPCR using standard amplification conditions. (B) qPCR of same template showing the effect of starting the PCR reaction with an extended 5 min (left panel) or 10 min (right panel) annealing/extension step. As can be seen increasing the annealing/extension time increases the ability of Taq polymerase to copy longer dU stretches. (C) Only the 5D4 blends are capable of amplifying the sulphonated templates. Interestingly, a higher 5D4 content in the blend results in an increased ability to copy the sulphonated residues as measured by real time PCR. Taq polymerase is unable to copy the sulphonated template even after a 10 min annealing/extension step.

In order to quantify and optimize the ability of 5D4 to promote PCR amplification of desulphonated DNA as well as evaluate its striking ability to PCR amplify non-desulphonated DNA, we performed qPCR amplifications of a 0.62 kb high content dC region (24.4% dC, region F, Figure 3) comparing standard PCR conditions with a modified PCR protocol comprising extended annealing and extension step in the first PCR cycle to promote read-through of homopolymeric template dU runs and remaining dhU6S adducts. We first examined a bisulfite-treated and desulphonated (BS1) template (Figure 4A). While the blends of 5D4 and Taq perform well even under standard conditions, increasing annealing and extension times (5 or 10 min) in the initial PCR cycle allows weak amplification from these templates even by Taq alone. However, Taq/5D4 blends at all ratios are always superior (Figure 4B). Presumably, the expanded extension time allows even Taq polymerase alone to copy past some of the dU stretches allowing subsequent PCR amplification.

We next examined PCR amplification of the same high GC region using a non-desulphonated plasmid, in which template dCs had been converted to dhU6S adducts (but not resolved to dUs). Neither Taq nor the 5D4-Taq blends yielded detectable amplification in qPCR under standard (short extension time) PCR conditions. However, an extended extension time (5/10 min) enabled amplification of the sulphonated templates by the 5D4-Taq blends, while Taq alone is unable to copy the sulphonated template to any detectable degree even with prolonged extension times (Figure 4C). Interestingly, while the ‘high Taq’ blends (comprising Taq/5D4 ratios of 10/1 and 5/1) outperformed a 1/1 Taq /5D4 blend on the desulphonated template under all PCR conditions, on the non-desulphonated template a higher 5D4 content was found to be beneficial resulting in an increased ability to copy the sulphonated DNA as measured by qPCR.

**Figure 5.**
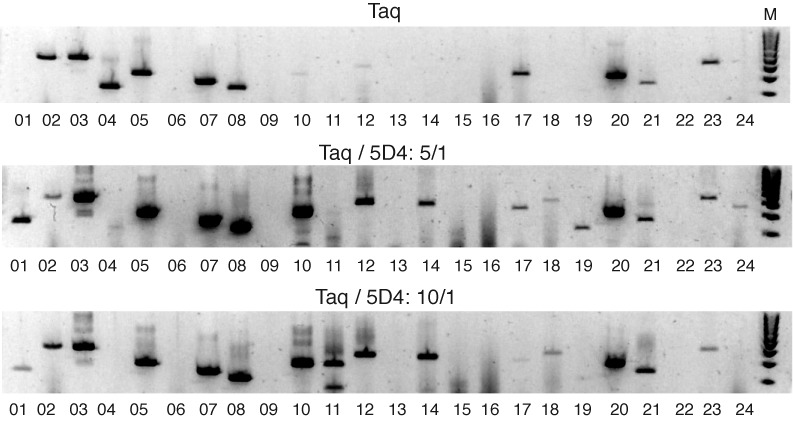
Amplification of 24 individual human genomic loci. Comparison of PCR performance of Taq (top) panel, with two different Taq/5D4 blends (5/1 (middle panel); 10/1 (bottom panel)) on the amplification of 24 different promotor regions in bisulfite-treated and fully desulphonated human genomic DNA. Both blends are able to amplify a significantly larger number of loci than Taq alone and together enable amplification of 18 out of 24 loci (75%).

Encouraged by the improved PCR activity of 5D4 on bisulfite-treated model plasmid templates, we wanted to test the ability of the enzyme to be integrated into a typical bisulfite sequencing workflow. To this we compared the ability of Taq/5D4 polymerase blends and Taq alone to generate PCR amplicons from diverse loci of bisulfite-treated human genomic DNA. Twenty-four human promoter regions were chosen at random and fully nested PCR primers (SI Supplementary Table S2) synthesized for each genomic region. We tested this approach using bisulfite-modified and fully desulphonated genomic DNA (BS1) from the liver cancer cell line HepG2 and a two-step nested PCR approach comparing Taq alone with two different Taq/5D4 blends (10/1 and 5/1) (Figure 5) in the first PCR. The second nested PCR amplification (25 cycles) used Taq only (since no remaining dU residues were present in the second round template) and was carried out in the presence of Syto-9 green fluorescent nucleic acid stain for quantification by real-time qPCR (Supplementary Figure S4). Again 5D4 exhibits enhanced bisulfite template amplification compared to Taq polymerase. The Taq/5D4 10/1 blend amplified 15 of the 24 genomic regions (62.5%), while the Taq / 5D4 5/1 blend performed even better amplifying 17 (71%) of the regions tested (Figure 5). This compares favourably with Taq, which yielded amplicons for only 12 (50%) of the regions tested. Indeed, together the blends perform even better amplifying 18 (75%) of the samples tested. In qPCR Taq/5D4 blends were outperforming or equalling Taq in qPCR (as judged by Ct values) in the majority of samples (Supplementary Figure S4). This data provides a stringent test of the ability of the 5D4 polymerase to increase sequence recovery from bisulfite-treated genomic DNA and indicates that addition of 5D4 DNA polymerase to a Taq-based PCR mix yields improved amplification even from a highly complex DNA mixture despite the increased primer degeneracy caused by bisulfite treatment.

**Figure 6.**
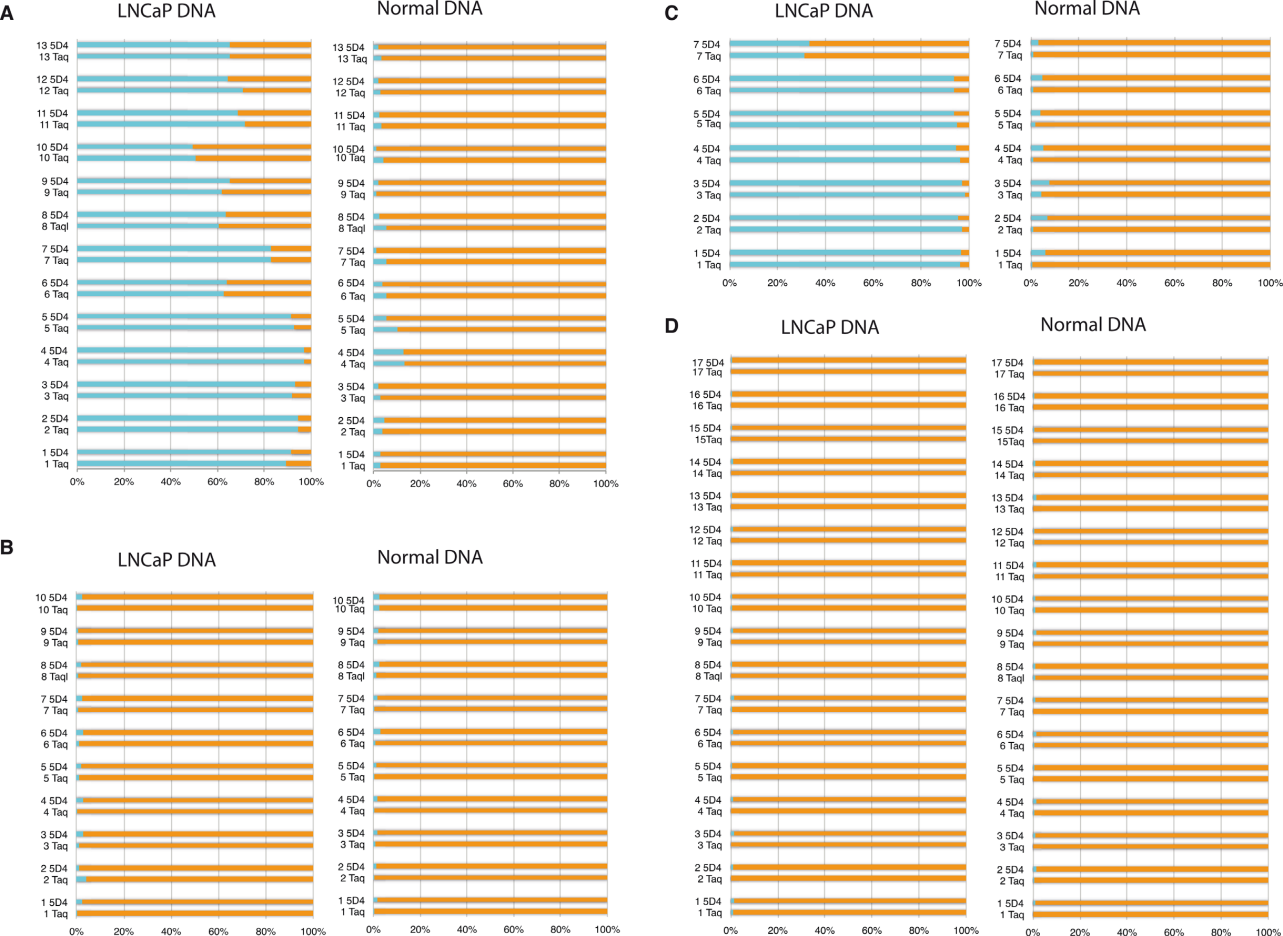
Degree of methylation of individual CpG sites. Promoter regions of four genes (**A**—*prkcdbp*, **B**—*dab2ip*, **C**—*ptgs2*,**D**—*ezh2)* were amplified with either Taq ot 5D4/Taq blends, using bisulfide-treated genomic DNA from normal cells or LNCapP cells as a template and subjected to deep sequencing. Cyan—methylated CpGs, orange—unmethylated CpGs. Individual CpGs are numbered starting from the 5′ end of the amplicon.

To rigorously assess the fidelity of Taq/5D4 blends in determining the methylation status we applied Illumina sequencing to amplicons derived from BS treated genomic DNA. We compared the methylation status of the promoter regions of four genes in normal human genomic DNA and genomic DNA derived from prostate cancer cell line LNCaP. The amplicons were selected in the promoter region of the genes *prkcdbp*, *dab2ip*, *ptgs2* and *ezh2* comprising a wide range of dC content and primers were designed to amplify both methylated and unmethylated sequence of BS treated DNA (Supplementary Table S5). A double nested PCR was performed for each amplicons, where the first PCR was performed by either Taq alone or by 5D4/Taq blends and the second by Taq alone. The resulting PCR products were subjected to deep sequencing using Illumina MiSeq V3–150 platform, yielding between 1–9 × 10^5^ high-quality reads of 117–120 bp (depending on locus) from the 5′-end of the each amplicon with uniformly high BS conversion (>99%) for most amplicons (Supplementary Table S3). The results were processed using www.galaxyproject.org (22–24). This allowed us to determine both the general degree of methylation and the methylation status at each individual CpG position of the amplicons with high confidence. These data revealed close agreement of the degrees of methylation as determined for amplicons derived by Taq alone and Taq/5D4 blends within a broad range of Taq/5D4 ratios, in particular in the cases of higher degrees of methylation (*prkcdbp* and *ptgs2* amplicons derived from LNCaP DNA). Deep sequencing data of amplicons generated by Taq and 5D4/Taq blends also show close agreement in methylation patterns at individual CpG sites, with amplicons, derived from LNCaP DNA showing a comparably high methylation of the individual CpGs of *prkcdbp* and *ptgs2* genes (Figure 6).

**Figure 7.**
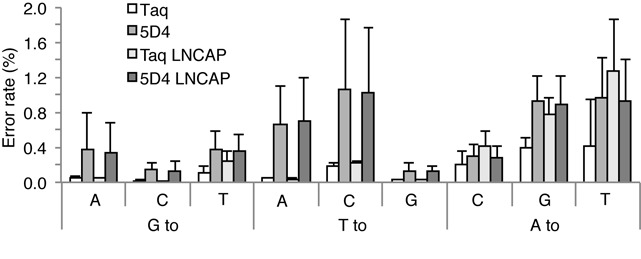
Breakdown of error rates by individual nucleotides. Data are presented as average +/−SD of the errors over the four amplicons (*prkcdbp*, *dab2ip*, *ptgs2* and *ezh2*) of bisulfite-treated genomic DNA. A breakdown of error rates by individual nucleotides for each amplicon is shown in Supplementary Tables S7 A–D. Only error rates of A, T and G are shown, as during bisulfite treatment C can be either converted to T or remain as C depending on its methylation status and the efficiency of BS conversion, which makes it impossible to assess the real error rate. For T only errors in the original Ts in the genomic DNA (no C to T conversions) are shown.

Regarding the general fidelity of 5D4/Taq blends (after a total of 70 cycles of PCR) we determined an error rate of 0.2–0.4% for Taq (3–6 × 10^−5^/cycle) and 0.8–1.2% (1–1.7 × 10^−4^/cycle) for the 5D4 blends (Supplementary Table S3). Breakdown of error rates by individual nucleotides showed similar trends (Figure 7 and Supplementary Table S7: A–D). To determine whether the higher error rate of Taq/5D4 blends is specific for the bisulfide treated templates, we also deep sequenced the *prkcdbp* promoter region from LNCaP cells as untreated genomic DNA. General fidelity and the breakdown of error rates by individual nucleotides of Taq/5D4 blends when amplifying unmodified genomic DNA were again similar to the fidelity of amplifying BS treated templates (Supplementary Tables S4 and S7E). We note that the reduced fidelity of Taq/5D4 blends does not affect the high confidence in determining the methylation status of individual CpG sites.

## DISCUSSION

Polymerases have been engineered for many different applications (25,26). Successful approaches include engineering by design (27), screening (28) and selection including phage display (29), compartmentalised self-replication (CSR) (18) and self-tagging (CST) (30). These methods have yielded a number of potentially useful polymerases including polymerases for next-generation sequencing, with an ability to amplify damaged DNA, utilize hydrophobic base analogues and fluorescent-dye modified bases and fully replace dNTPs with unnatural synthetic analogues (16,17,19,27,30). Here we describe the characterization of the utility of one such polymerase (5D4), originally selected for bypass of hydrophobic base analogues (16) for PCR amplification of bisulfite-treated DNA with potential applications in epigenomics.

5D4 is a Tth/Taq chimera with 14 additional mutations from the Taq consensus (V62I, Y78H, T88S, P114Q, P264S, E303V, G389V, E424G, E432G, E602G, A608V, I614M, M761T, M775T), which was originally selected for bypass of hydrophobic base analogues using the non-hydrogen bonding base analogue 5-nitroindol (d5NI) as bait (16). Previous analysis of mutations and the chimeric polymerase structure of 5D4 suggested that the Tth 5′-3′ exonuclease domain and resident mutations as well as the A608V mutation in the main polymerase domain were selected mostly for reasons of thermostability. Four mutations (E602G, I614M, M762T and M775T) in the main polymerase domain were conserved among other selected polymerases displaying a 5D4-like phenotype and therefore are likely to be connected to function, with I614M found to make the key contribution. I614 is located in the A-motif within the polymerase active site and is directly involved in binding the incoming dNTP substrate. Mutations at I614 (or equivalent residues in other polymerases) and their phenotypic effects have been described multiple times in the literature. They have been found, for example, to relax discrimination against ribonucleotides (20,31) and other non-canonical substrates. The change from Ile to Met, reduces steric bulk in the active site and may relax geometric constraints on substrate selection. Furthermore, statistical coupling analysis suggested a connection of the M762T and M775T mutations to F667, another active site residue making close contacts with the incoming dNTP. Both of these mutations appear to be responsible for a further reduction of steric constraints and therefore a more relaxed geometric substrate selection in the 5D4 active site.

d5NI causes significant geometric distortion of the primer–template duplex by intercalation into the opposing strand base-stack, yet 5D4 is able to replicate across both d5NI homo- and heteropairs with the natural bases (16). It may be this tolerance to geometric distortions that enables 5D4 to deal with the aberrations from planar geometry and correct stacking interactions caused by DNA lesions or residual bisulfite adducts (such as dhU6S). In the latter case, comparison with the structurally related dihydrouridine (dhU) found in tRNAs may be illustrative. dhU is known to destabilize tRNA structure by disrupting stacking interactions due to a puckering of the pyrimidine ring of 0.47 Å at C6, as well as by affecting the ribose ring conformation. dhU6S also forms two different diastereoisomers at the C6 centre and it is unknown if both can serve as a templating base for polymerases. Nevertheless, it is clear that 5D4 has a much-enhanced ability to bypass template dhU6S compared to Taq and this is particularly evident on homopolymeric runs (Figure 2). What is more, 5D4 is able to decode the dhU6S template base correctly, predominantly inserting dATP (as with dU) (Supplementary Figure S1). However, the enhanced ability of the 5D4 DNA polymerase to copy and amplify bisulfite-treated DNA not only arises from its superior ability to ‘read’ across the distorting dhU6S adduct but, more importantly, from an enhanced ability to utilize the non-canonical dU as a templating base much more efficiently than Taq polymerase. Polymerase stalling and inhibition in response to template dU is well known from polymerases of the polB family from hyperthermophilic archaea (15) and is thought to be an adaptation to the increased rate of dC deamination at high temperatures. Although bacteria of the genus *Thermus* including *T. aquaticus*, from which Taq DNA polymerase derives, grow at slightly lower temperatures (70°C), one might speculate that a similar (although less stringent) mechanism might bias its substrate spectrum. Alternatively, the poor utilization of template dU by Taq may simply be based on functional differences between dT and dU, such as weaker stacking and base-pairing with dA of the latter. Finally, part of the improved ability of 5D4 to amplify bisulfite-treated DNA may also be related to its generally enhanced ability to bypass lesions in the DNA template strand, as shown earlier (16). Such lesions may occur more frequently in bisulfite-treated DNA due to the harsh conditions of desulfonation.

We had previously examined the fidelity of 5D4 on unmodified DNA and had found that the rate of nucleotide misincorporation (3.1 × 10^−4^) by 5D4 is increased ca. five-fold compared to Taq (16). Indeed, we find a comparable fidelity difference for Taq/5D4 blends as determined by deep sequencing of amplification products. It may be noted that this slightly reduced fidelity does not compromise the correct readout of methylation status at relevant positions but rather appears as a background noise in the known sequence context.

In summary, we describe 5D4, an engineered DNA polymerase, which displays a greatly enhanced ability to PCR amplify bisulfite-treated DNA (Figures 3–5) in particular from regions of high GC content, which promises efficiency and sensitivity gains in bisulfite sequencing. Its activity is entirely compatible with standard PCR protocols (using Taq) and can be integrated seamlessly into current bisulfite sequencing workflows. It is remarkable that the enhanced activity of 5D4 on bisulfite-treated DNA is an unintended (and unexpected) consequence of its original selection purpose. We anticipate that considerable further gains in efficiency and sensitivity could be realized by direct selection of 5D4 for amplification of bisulfite-treated DNA.

## Supplementary Material

SUPPLEMENTARY DATA
