# Supplementary material for: A polymerase engineered for bisulfite sequencing
Source: Nucleic Acids Res. 2015 Aug 13;43(22):e155. doi: 10.1093/nar/gkv798 (PMC4678845; doi:10.1093/nar/gkv798)
Supplement: SUPPLEMENTARY DATA [file supp_43_22_e155__index.html]

A polymerase engineered for bisulfite sequencing — A polymerase engineered for bisulfite sequencing — SUPPLEMENTARY DATA 

# A polymerase engineered for bisulfite sequencing

## SUPPLEMENTARY DATA

- SUPPLEMENTARY DATA
