# Supplementary material for: A polymerase engineered for bisulfite sequencing
Source: Nucleic Acids Res. 2015 Aug 13;43(22):e155. doi: 10.1093/nar/gkv798 (PMC4678845; doi:10.1093/nar/gkv798)
Supplement: SUPPLEMENTARY DATA [file supp_gkv798_nar-01550-met-k-2015-File009.pdf]

Supporting information:

A polymerase engineered for bisulfite sequencing

Doug Millar[1] Yonka Christova[2] and Philipp Holliger[2]

Address: [1]Genetic Signatures, Sydney, Australia; [2]MRC Laboratory of Molecular Biology, Francis Crick Avenue, Cambridge Biomedical Campus, Cambridge CB2 0QH, UK

Supporting Figures: S1-S4

Supporting Tables S1-S7

Supporting Figures

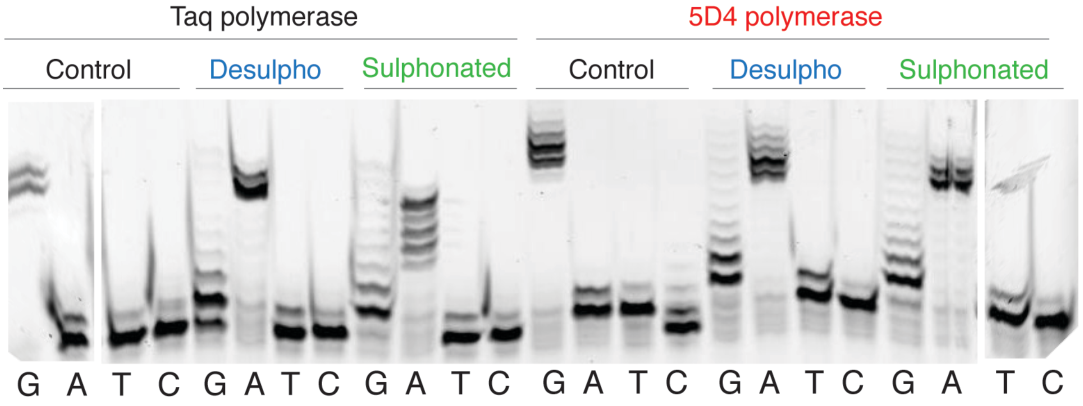

Fig. S1 Base insertion fidelity opposite dU and dhU6S
Base insertion fidelity of DNA polymerase Taq and 5D4 on template T2 (dC_8) either umodified (Control), bisulfite treated and desulfonated (Desulpho: Reagent 1, 80°C, 20min) (converting dC_8 to dU_8) or bisulfite treated (Reagent 1) but not desulphonated (converting dC_8 to $dhU6S_8$) (Sulphonated). Upon addition of either dGTP, dATP, dTTP or dCTP (G, A, T, C) (5 min extension) both polymerases correctly insert dGTP on control template dC_8. We also observe some misincorporation of dATP and dTTP opposite dC by 5D4 and to a lesser extend dATP by Taq, most likely due to non-specific extendase (non-templated nucleotide addition) activity of both polymerases. Upon bisulfite treatment and desulphonation both polymerases now predominantly insert dATP as dC_8 has been converted to dU_8. On the bisulfite treated but non-desulphonated template both polymerases read $dhU6S_8$ as dU inserting predominantly dATP. However, Taq is unable to generate full-length extension product indicating inhibition by the sulfonate adduct. Note also some extension upon dGTP addition indicating some incompleteness to bisulfite conversion

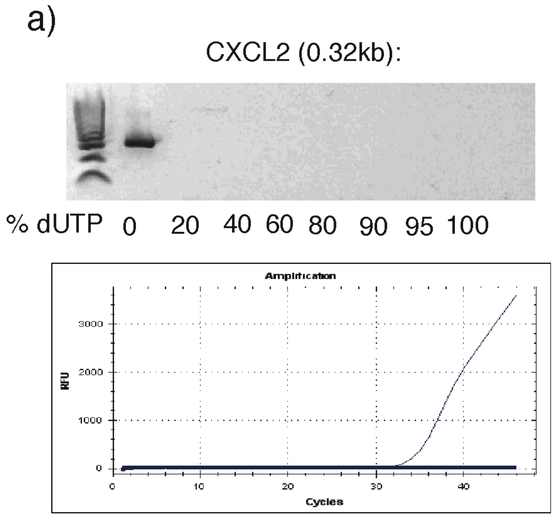

	mM dTTP	mM dUTP	Ct value
B01	10	0	31.26
B02	8	2	N/A
B03	6	4	N/A
B04	4	6	N/A
B05	2	8	N/A
B06	1	9	N/A
B07	0.5	9.5	N/A
B08	0	10	N/A
B09	neg		N/A

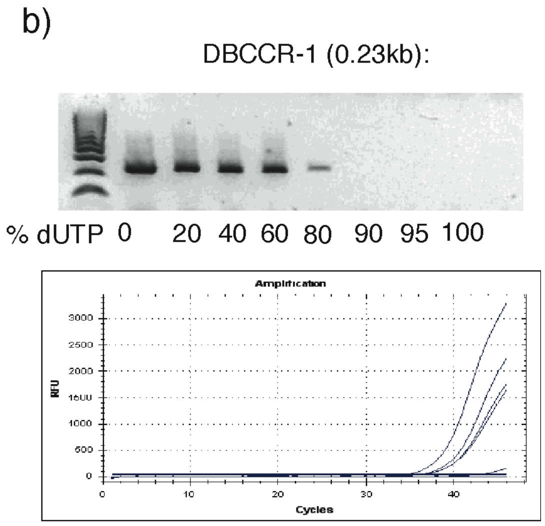

	mM dTTP	mM dUTP	Ct value
C01	10	0	34.03
C02	8	2	35.66
C03	6	4	35.97
C04	4	6	36.06
C05	2	8	42.46
C06	1	9	N/A
C07	0.5	9.5	N/A
C08	0	10	N/A
C09	neg		N/A

Fig. S2 The effects of dUTP in PCR amplification using Taq polymerase
PCR amplification as a function of dUTP content in the otherwise standard PCR mix (from 0% dUTP to 100% dUTP) and amplifying a 0.32 kb fragment from the chemokine (C-X-C motif) ligand 2 (CXCL2) and a 0.23 kb fragment from the deleted in bladder cancer 1 (DBCCR1) promoter region (Table S2, primers 2 and 3). The two genomic regions varying in total C content (26.6% vs 18.7% respectively) see earlier comment in the main text. Top panels, show agarose gel electrophoresis of PCR bands, middle panel show qPCR curves and bottom panel, qPCR Ct values for individual reactions.

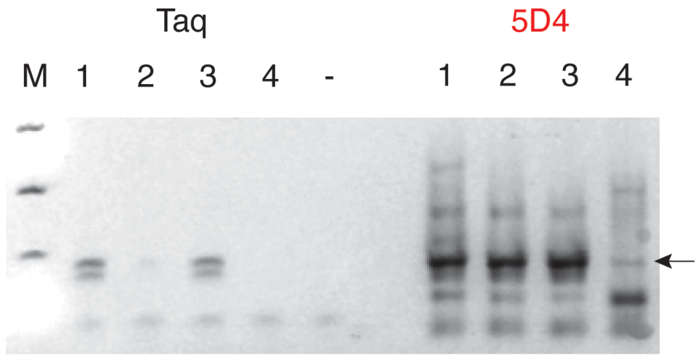

Figure S3 PCR amplification of desulphonated and non-desulphonated DNA
Comparison of PCR amplification by Taq (left panel) vs 5D4 DNA polymerase (right panel) from plasmid DNA treated with bisulfite according to different protocols varying the degree of desulphonation and DNA damage (1 (Buffer 1, 10 min 95°C); 2 (Buffer 1, no heat treatment), 3 (Buffer 2 (high pH), no heat treatment), 4 (Water control, no heat treatment).

a)

Taq | Taq/ 5D4 5/1 | Taq/ 5D4 10/1

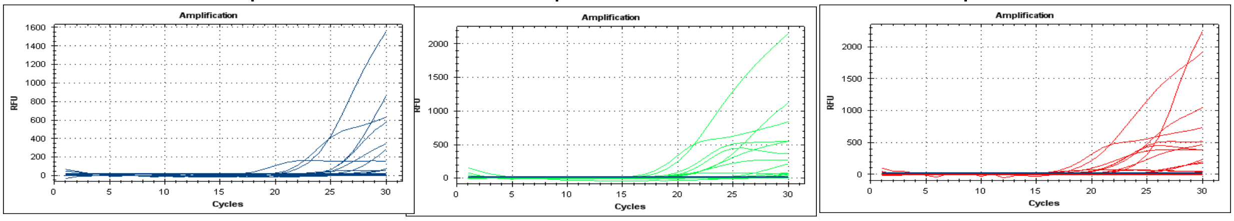

b)

Taq / 5D4	01	02	03	04	05	06	07	08	09	10	11	12
10/1	n/a	19.62	20.14	n/a	18.72	n/a	16.41	18.62	n/a	18.11	21.12	23.92
5/1	n/a	21.53	18.91	n/a	18.66	n/a	16.26	19.07	n/a	17.61	n/a	24.35
Taq	n/a	16.7	23.51	23.11	23.2	n/a	19.98	25.12	n/a	n/a	n/a	n/a
	13	14	15	16	17	18	19	20	21	22	23	24
10/1	n/a	24.42	n/a	n/a	n/a	n/a	n/a	15.31	23.87	n/a	29.62	n/a
5/1	n/a	27.3	n/a	n/a	n/a	n/a	n/a	15.4	25.4	n/a	28.08	n/a
Taq	n/a	n/a	n/a	n/a	26.01	n/a	n/a	19.47	29.06	n/a	27.74	n/a

Figure S4 qPCR amplification of 24 individual human genomic loci
a) qPCR traces (from left to right) for Taq only (blue) as well as for Taq/ 5D4 blends 5/1 (green) and 10/1 (red). b) qPCR Ct values for this experiment are tabulated. Samples 1, 18 and 19 were not reliably detected as the melting temperature from these particular amplicons was just below the data collection temperature (75°C). Among the other samples Taq / 5D4 blends outperform Taq alone for samples 3, 5, 7, 8, 10-12, 14, 20 and 21 (red), while Taq outperforms the blends for samples 2, 4 and 17 (blue).

Supporting Tables

Table S1: Oligonucleotides (Primers / templates)

Templates / primers	Sequence (5'-3')
Extension	Cy3-CT$_{LNA}$CA$_{LNA}$CTATACCCA
Template T1	TAGCACCCTAGCCAGCTAGCTGGGTATAGTGAGTGGT
Template T2	CCCCCCCCTGGGTATAGTGAGTGGTATTA
Template T3	UUUUUUUUTGGGTATAGTGAGTGGTATTA

Table S2: Gene list & Primer sequences

Gene	size (bp)	Genebank	Primers (5'-3') (#1, #4, #2, #3)
mdr1	324	L07624	GTATTTGTATTTTGAAATATTTGTT, AAATACTAATTCCTCTAAAAACTAC, GTAATTTAGAAATTGTTAAGTATGT, CAAATTAAATTTCCAAAAAAAAATATTC
brca1	457	AY273801	GGGGGTAGATTGGGTGGTTAATTT, ATCAAACTAATCTAAAACTCCTAACC, GGTTGTTGTTTAGAGGTAGTTTTTTGG, AACATAATATCCCCCTCAAAACATATTCCAA
casp8	457	AY291598	ATAGTTTTTTATATTGTAATTTAAGGTAATGT, ACAAACAACAAATACTCCAAAAATA, GTATAAAGGGATGAGTTAGATTGT, ACTAATATTCCTCAAACAACCACCAATCAC
chfr	250	NW_004078076	GGATTTGTGTGATTTATTGTGTGT, CAAAAAAACATTACTACTCCCTCAAC, GGGGTTTTTAGAATTTTTGGGGTTTT, AAACTAAACCAAAAACCTCTAC
cxcl2 (mgsa-beta)	320	U03019	ATTGAAATGTTTTTTAGAGAAGTAATTTT, AACTAACAAAAAACTACCTATAACC, GTGGGTTTAAGGGATTTGATTT, ACCCCTTTTATACATAATTAAAAC
bcl-2	407	NM_000657	TTTTTTAGTATTATAGAGGAAGTAGAT, TCAAATACAACTAACTAAACATCTC, TTATGTTAAGGGGGAAATATTAGAATT, AATCTACAACTACTAAATCCTAAC
dapk1	276	NT_008470.19	ATTAAGTATAGTGTTAGGTATAAGT, AAACAACCTCTCTACTCCTTAC, GTTTTTTTAGAATTTAGTTAGAGGGT, CAAACTACCAATAAAAACCCTACAAAC
dbccr1	432	NM_014618	GATAAAATTTATATTACATGGATTT, CTAATCCCTAAAACAAACATAAATCC, TATAGTATAGTTAAGTTTAAGGGTATT, CAAATCAATTTACAACCTTAAAATCC
dlc-1	398	AF51429	GTTTAAGGTATATTAGGGTTTAGGT, CTCAAAAAAATTAAACTAAAAATCC, GTTTTTAGTTGGGAAAGTGGAGGTT, AAAAAATACTACCATATCCTAAC

epb41	368	BC006141	GAGAGGGGTTTGGAGAGGTTAGGTT,<br>CCCTCTCAATCCCCCACTCCTAAAAAC,<br>GTTAAAGTTATTGGTATTGGTAGTTGTT,<br>TACCTAAAATCAACAAAAAACC
hox-1.3	388	M26679	GTTTATTTTTAGAGGTTATTAGGT,<br>TCTCTACTACTAATATAAATACTAC,<br>GAGGGAATTAAGTATATGTTTTAGTT,<br>CTTAACCTACTAACTATACCTAAAC
hoxa11	400	AF071164	GAAAGAGGATTTTGTAATTGAGTAT,<br>CTTAACAAATACATATTAAAAAAAC,<br>TATGTTGTATGGGGGAGTATTTTAGTT,<br>TTCATTTATTAAACTACCTTAAAC
hic1	375	L41919	GGTAATTGTTTTTAAAAGGGTTAT,<br>ATATAAATAAAATCCAACACCAAACTAAAC,<br>GTTTTTATTTTAGAGGGTAGTTGG,<br>ATTAAACTAATTATCATACACCACCAAAA
igfbp7	432	AY518539	TGAGTATTTAGGTGTTTTAGGTTT,<br>CAAAAACAAAAACAACAACCCAAC,<br>GAGGATTTAATAGATGAAATTT,<br>CTCCATAACTAAATACTATAACAAC
igf2	371	AF517226	GTTTTTTTTGGATAATTAGAAGAATTT,<br>TACCCAACACCCCTAAAACCAAAAC,<br>TTGGTAGAGGAGTGTTAGGTAGGAGGGTT,<br>CATATAAAACTACACCCCCTACAC
magea3	339	U03735	AGAGGATGGAGGTTTAGGTTTAGTT,<br>CCCAACCTAAAAATCTTCCCCTAC,<br>ATTAGGATTTATAGTTTTAGGATT,<br>ACAACACTAAATTATTTAAAACCC
mgmt	391	X61657	GATTATTTTTGTGATAGGAAAAGGTAAGGGTTAAAA<br>ACTAAACTACCCAAACACTCACCAAATC<br>ATTTGGTAAATTAAGGTATAGAGTTTTAGG<br>TTCTAAAAACTCTAACTATCCCAA
muc-1	246	NW_004077999	AAAGTTTTTAGTYGTTTATTATGT<br>AAACCCTAAAATCCTTCCTTAC<br>TTTGGTTAYGGTAGTGATTT<br>CCACTTAAAAAAATAAACCTAAAC
psen1	359	NW_004078079.1	GATGTAGTTGGTTYGTAAATAGGTATTT<br>AATTCCTTCCAAACCAACCACTAT<br>TAGGTGTTAAATTAGGATGGTT<br>AAACTACCTATCCCAAAACCC
rassf1	329	AC002481	GGTTTTATAGTTTTTGTATTTAGGT<br>CCCCAAATAAAATCTCCACAAAAA<br>TTTAGTTTGGATTTTGGGGGAGGAGT<br>TAACCACTACCAAAAACCAACTAC
rfc1	192	NW_004078018	GTAATTGGGAGGTATTTGGGTATTT<br>AAAATCTATTATCRAAACTCAAAATC<br>TTAAGGAAATTAATATYGGATTTTTTTAT<br>TACAAAAAAACRAAAACAATTAA
rarb	412	X56849	TTAAGGGGTAGTTATTTTTTGTTTTATAG<br>CAAACAACTCTCATAAAAAAATTCTAA<br>TAGGAGGAATATAGTTTTTTAAGTTAAGT<br>AACAAAAAATAAACCCTCCTACCTCTAAAC
serpinb5	362	BC020713	GGTATTTAGTAGAATGAGTTGTTGT<br>CACAAAAAACCTAAATATAAAAAC<br>GGAGATTAGAGTATTTTTTGTTTT<br>ACAACTATAACTCACCTAAACAAC

Table S3: Deep sequencing of amplicons derived by Taq or Taq/5D4 blends

Gene	Polymerase and template	Number of reads	% BS conversion	% error	% methylation
prkcdbp	Taq Normal	241798	99.66	0.36	3.16
	5D4 Normal	427553	99.03	1.12	4.91
	Taq LNCaP	211123	99.73	0.27	65.47
	5D4 LNCaP	113028	99.27	0.95	67.39
dab2ip	Taq Normal	649236	99.19	0.42	1
	5D4 Normal	621619	96.94	1.68	2.08
	Taq LNCaP	971115	99.72	0.16	0.86
	5D4 LNCaP	431308	99.12	1.14	1.98
ptgs2	Taq Normal	205995	99.62	0.3	1.64
	5D4 Normal	200736	98.37	1.3	5.33
	Taq LNCaP	236719	99.69	0.8	86.82
	5D4 LNCaP	163387	98.54	1.27	86.34
ezh2	Taq Normal	461731	99.58	0.44	0.41
	5D4 Normal	487044	99.17	1.14	0.27
	Taq LNCaP	571859	99.68	0.42	1
	5D4 LNCaP	446390	99.24	1.13	0.89

Table S4: Error rate of Q5, Taq and Taq/5D4 for unmodified genomic DNA.

Polymerase	[1]Error rate (%)
Q5	0.05
Taq	0.10
5D4/Taq 1:10	0.80
5D4/Taq 1:20	0.78

[1]*Error rates after 70 cycles of PCR (as in Table S3). Template: unmodified genomic DNA, isolated from LNCaP cells. Amplicon: promoter region of prkcdbp gene.*

Table S5: List of primers for Illumina sequencing[1]

Gene	Amplicon size	Primers
prkcdbp	453	GATTTGGGGTTAATAGGTTTTTTAGT
		TTGGTTTTTATGTTGGAGATTTTG
dab2ip	238	GGGGAGTTATTAAGTTTAGGTAGT
		GTAATAGTTAGTTGGTGAGTAGT
ptgs2	498	TTAAGGTGATTAGTTTAGAATTGGTTT
		TAGGAGTATGTTTAGGAATTTTTTAGTAG
ezh2	208	GGTTTAAATTTGGTTTTTAGT
		GTTTGTATATTGTTTTTTTGAGAGG

[1]*Underlined TGs denote CpGs in the unmodified genomic sequence*

Table S6: List of Illumina adaptors

Gene	Templ	Pol	Sequence (5', 3')
prkcdbp	normal	Taq	AATGATACGGCGACCACCGAGATCTACACTCTTTCCCTACACGACGCTCTTC CGATCTNNNATCACGGATTTGGGGTTAATAGGTTTTTAGT
prkcdbp	LNCAP	Taq	AATGATACGGCGACCACCGAGATCTACACTCTTTCCCTACACGACGCTCTTC CGATCTNNNCGATGTGATTTGGGGTTAATAGGTTTTTAGT
prkcdbp	normal	5D4/Taq	AATGATACGGCGACCACCGAGATCTACACTCTTTCCCTACACGACGCTCTTC CGATCTNNNTTAGGCGATTTGGGGTTAATAGGTTTTTAGT
prkcdbp	LNCAP	5D4/Taq	AATGATACGGCGACCACCGAGATCTACACTCTTTCCCTACACGACGCTCTTC CGATCTNNNTGACCAGATTTGGGGTTAATAGGTTTTTAGT
dab2ip	normal	Taq	AATGATACGGCGACCACCGAGATCTACACTCTTTCCCTACACGACGCTCTTC CGATCTNNNGTGAAAGGGAGTTATTAAGTTTAGGTAGT
dab2ip	LNCAP	Taq	AATGATACGGCGACCACCGAGATCTACACTCTTTCCCTACACGACGCTCTTC CGATCTNNNACAGTGGGGAGTTATTAAGTTTAGGTAGT
dab2ip	normal	5D4/Taq	AATGATACGGCGACCACCGAGATCTACACTCTTTCCCTACACGACGCTCTTC CGATCTNNNCAGATCGGGAGTTATTAAGTTTAGGTAGT
dab2ip	LNCAP	5D4/Taq	AATGATACGGCGACCACCGAGATCTACACTCTTTCCCTACACGACGCTCTTC CGATCTNNNACTTGAGGGAGTTATTAAGTTTAGGTAGT
ptgs2	normal	Taq	AATGATACGGCGACCACCGAGATCTACACTCTTTCCCTACACGACGCTCTTC CGATCTNNNGATCAGTTAAGGCGATTAGTTTAGAATTGGTTT
ptgs2	LNCAP	Taq	AATGATACGGCGACCACCGAGATCTACACTCTTTCCCTACACGACGCTCTTC CGATCTNNNTAGCTTTTAAGGCGATTAGTTTAGAATTGGTTT
ptgs2	normal	5D4/Taq	AATGATACGGCGACCACCGAGATCTACACTCTTTCCCTACACGACGCTCTTC CGATCTNNNGGCTACTTAAGGCGATTAGTTTAGAATTGGTTT
ptgs2	LNCAP	5D4/Taq	AATGATACGGCGACCACCGAGATCTACACTCTTTCCCTACACGACGCTCTTC CGATCTNNNCTTGTATTAAGGCGATTAGTTTAGAATTGGTTT
ezh2	normal	Taq	AATGATACGGCGACCACCGAGATCTACACTCTTTCCCTACACGACGCTCTTC CGATCTNNNAGTCAAGGTTTAAATTTGGTTTTTAGTAT
ezh2	LNCAP	Taq	AATGATACGGCGACCACCGAGATCTACACTCTTTCCCTACACGACGCTCTTC CGATCTNNNAGTTCCGGTTTAAATTTGGTTTTTAGTAT
ezh2	normal	5D4/Taq	AATGATACGGCGACCACCGAGATCTACACTCTTTCCCTACACGACGCTCTTC CGATCTNNNATGTCAGGTTTAAATTTGGTTTTTAGTAT
ezh2	LNCAP	5D4/Taq	AATGATACGGCGACCACCGAGATCTACACTCTTTCCCTACACGACGCTCTTC CGATCTNNNCCGTCCGGTTTAAATTTGGTTTTTAGTAT
prkcdbp	N/A	N/A	CAAGCAGAAGACGGCATACGAGATCGGTCTCGGCATTCCTGCTGAACCGCT CTTCCGATCTCAAAATCTCCAACATAAAAACCAA
dab2ip	N/A	N/A	CAAGCAGAAGACGGCATACGAGATCGGTCTCGGCATTCCTGCTGAACCGCT CTTCCGATCTACTACTCACCAACTAACTATTAC
ptgs2	N/A	N/A	CAAGCAGAAGACGGCATACGAGATCGGTCTCGGCATTCCTGCTGAACCGCT CTTCCGATCTCTACTAAAAAATTCCTAAACATACTCC
ezh2	N/A	N/A	CAAGCAGAAGACGGCATACGAGATCGGTCTCGGCATTCCTGCTGAACCGCT CTTCCGATCTCCTCTCAAAAAAACAATATACAAAC

Table S7: Breakdown of error rates by individual nucleotides for each amplicon. Error rates are derived by deep sequencing of bisulfide-treated DNA of the promoter region of *prkcdbp* (A), *dab2ip (B), ptgs2 (C) and ezh2* (D) and untreated DNA of the promoter region of *prkcdbp*. See also **Fig. 7**

**A**		**BS treated PRKCDBP**			
		Taq normal	5D4 normal	Taq LNCaP	5D4 LNCaP
G to	A	0.046	0.196	0.039	0.162
	C	0.028	0.115	0.019	0.077
	T	0.068	0.229	0.041	0.180
T to	A	0.039	0.461	0.037	0.437
	C	0.128	0.555	0.130	0.607
	G	0.016	0.072	0.009	0.061
A to	C	0.263	0.270	0.184	0.245
	G	0.310	0.665	0.268	0.625
	T	0.186	0.649	0.097	0.551

**B**		**BS treated DAB2IP**			
		Taq normal	5D4 normal	Taq LNCaP	5D4 LNCaP
G to	A	0.043	0.153	0.043	0.195
	C	0.008	0.100	0.008	0.094
	T	0.052	0.221	0.058	0.269
T to	A	0.056	0.490	0.063	0.526
	C	0.141	0.786	0.158	0.640
	G	0.027	0.113	0.027	0.098
A to	C	0.153	0.247	0.174	0.216
	G	0.303	0.725	0.369	0.715
	T	0.188	0.562	0.115	0.542

**C**		**BS treated PTGS2**			
		Taq normal	5D4 normal	Taq LNCaP	5D4 LNCaP
G to	A	0.073	1.017	0.074	0.857
	C	0.010	0.269	0.012	0.290
	T	0.057	0.687	0.038	0.641
T to	A	0.039	1.326	0.046	1.433
	C	0.223	2.265	0.185	2.155
	G	0.026	0.266	0.020	0.214
A to	C	0.044	0.220	0.048	0.193
	G	0.404	1.259	0.413	1.346
	T	0.029	1.086	0.035	1.079

**D**		**BS treated EZH2**			
		Taq normal	5D4 normal	Taq LNCaP	5D4 LNCaP
G to	A	0.037	0.161	0.042	0.164
	C	0.009	0.117	0.009	0.074
	T	0.236	0.368	0.248	0.338
T to	A	0.037	0.360	0.035	0.401
	C	0.214	0.672	0.214	0.702
	G	0.027	0.096	0.023	0.102
A to	C	0.391	0.487	0.410	0.474
	G	0.552	1.067	0.774	0.882
	T	1.224	1.575	1.270	1.536

**E**		**PRKCDBP**		
		**Unmodified genomic LNCaP DNA template**		
		Taq unmodified	5D4 unmodified	Q5 unmodified
G to	A	0.034	0.312	0.022
	C	0.016	0.058	0.003
	T	0.013	0.248	0.022
T to	A	0.024	0.340	0.023
	C	0.102	0.488	0.028
	G	0.004	0.066	0.003
A to	C	0.091	0.146	0.073
	G	0.098	0.658	0.011
	T	0.021	0.412	0.008
C to	A	0.026	0.255	0.021
	G	0.004	0.091	0.004
	T	0.045	0.322	0.012
